# Untangling the Molecular Interactions Underlying Intracellular Phase Separation Using Combined Global Sensitivity Analyses

**DOI:** 10.1007/s11538-024-01288-y

**Published:** 2024-04-20

**Authors:** Kelsey I. Gasior, Nicholas G. Cogan

**Affiliations:** 1 Department of Applied and Computational Mathematics and Statistics, University of Notre Dame, Notre Dame, IN USA; 2 Department of Mathematics, Florida State University, Tallahassee, FL USA

**Keywords:** Liquid-liquid phase separation, Sensitivity analysis, Morris method screening, Sobol’ sensitivity analysis

## Abstract

Liquid-liquid phase separation is an intracellular mechanism by which molecules, usually proteins and RNAs, interact and then rapidly demix from the surrounding matrix to form membrane-less compartments necessary for cellular function. Occurring in both the cytoplasm and the nucleus, properties of the resulting droplets depend on a variety of characteristics specific to the molecules involved, such as valency, density, and diffusion within the crowded environment. Capturing these complexities in a biologically relevant model is difficult. To understand the nuanced dynamics between proteins and RNAs as they interact and form droplets, as well as the impact of these interactions on the resulting droplet properties, we turn to sensitivity analysis. In this work, we examine a previously published mathematical model of two RNA species competing for the same protein-binding partner. We use the combined analyses of Morris Method and Sobol’ sensitivity analysis to understand the impact of nine molecular parameters, subjected to three different initial conditions, on two observable LLPS outputs: the time of phase separation and the composition of the droplet field. Morris Method is a screening method capable of highlighting the most important parameters impacting a given output, while the variance-based Sobol’ analysis can quantify both the importance of a given parameter, as well as the other model parameters it interacts with, to produce the observed phenomena. Combining these two techniques allows Morris Method to identify the most important dynamics and circumvent the large computational expense associated with Sobol’, which then provides more nuanced information about parameter relationships. Together, the results of these combined methodologies highlight the complicated protein-RNA relationships underlying both the time of phase separation and the composition of the droplet field. Sobol’ sensitivity analysis reveals that observed spatial and temporal dynamics are due, at least in part, to high-level interactions between multiple (3+) parameters. Ultimately, this work discourages using a single measurement to extrapolate the value of any single rate or parameter value, while simultaneously establishing a framework in which to analyze and assess the impact of these small-scale molecular interactions on large-scale droplet properties.

## Introduction

A mechanism for intracellular organization is liquid-liquid phase separation (LLPS)  (Langdon et al. 2018; Elbaum-Garfinkle et al. 2015). Occurring in both the cytoplasm and the nucleus, these membrane-less compartments are vital to normal cellular function (Nott et al. 2015; Feric et al. 2016). Driven by LLPS, molecular complexes form and coalesce into dynamic liquid-like droplets, concentrating proteins and RNAs within the cell and promoting their interactions  (Nott et al. 2015; Weber and Brangwynne 2012; Brangwynne et al. 2015). The structure of the proteins and RNAS involved in LLPS is such that multivalent interactions can occur between the molecules  (Weber and Brangwynne 2012; Berry et al. 2018). Further, these proteins can interact and bind with different RNA species within the cellular environment  (Lee et al. 2013, 2015). Depending upon the RNA species involved, the resulting protein-RNA complex can create droplets with different physical properties, intracellular locations, and cellular functions. A prime example is in the multinucleated fungal cell, *Ashbya gossypii*, where a single protein species can have multivalent interactions with several different RNA species. Depending upon the RNA-binding partner, the resulting droplets can control different cellular functions, such as nuclear division or polarity  (Lee et al. 2013, 2015; Zhang et al. 2015). However, *in vitro* experiments have shown that, despite sharing a common protein-binding partner, droplets formed through the interactions with one RNA species do not colocalize with those formed from a different species. Instead, these different droplets are capable of coexisting and produce a heterogeneous droplet field  (Langdon et al. 2018).

The mechanisms underlying intracellular phase separation have been the subject of many different mathematical models. Previous work by Gasior and Zhao et al. created a modeling framework to understand the multivalent interactions between proteins and RNAs driving phase separation in *Ashbya gossypii* (Gasior et al. 2019). In this work, the authors examined the interactions between a single protein species and a single RNA species capable of bivalent interactions. Here, an RNA molecule could bind with one or two protein molecules to create two potential protein-RNA complexes: a monovalent protein-RNA complex or a bivalent protein-RNA-protein complex. Under *in vitro* initial conditions, these two complexes competed for a limited pool of free protein to form, but worked cooperatively to drive phase separation. Using a Cahn-Hilliard diffuse interface model  (Cahn and Hilliard 1958), coupled with a double-well chemical potential, mass action kinetics, and phase-dependent diffusion, this work studied how the competition between the monovalent and bivalent protein-RNA complexes impacted initial phase separation and droplet field evolution. Ultimately, Gasior and Zhao et al. found that the competition for free protein between cooperating protein-RNA species solely influenced the time scale of phase separation, but the coupling of these dynamics with intradroplet viscosity controlled intradroplet organization and evolution.

Work by Gasior et al. (2020) also studied protein-RNA interactions driving LLPS in *Ashbya gossypii*. While Gasior and Zhao et al. focused on the bivalent dynamics between a single protein and single RNA species, the work by Gasior et al. focused on multiple RNA species competing for the same protein-binding partner necessary for driving phase separation. This work used the Cahn-Hilliard diffuse interface model paired with mass action kinetics and phase-dependent diffusion. However, Gasior et al. focused on monovalent protein-RNA interactions and used the Flory-Huggins (FH) free energy scheme, rather than a double well chemical potential, in their model. Using *in vitro* initial conditions, this work studied how competition between different RNA species influenced phase separation and the composition of the droplet field. Ultimately, the authors determined that the competition between the two RNA species for free protein dictated droplet field composition at the onset of separation.

While the conclusions reached by Gasior and Zhao et al. and Gasior et al. shed light on the influence of certain biological parameters on experimentally measurable values, their results are lacking in scope due to simplifications in analysis. The competition between the monovalent and bivalent protein-RNA interactions  (Gasior et al. 2019) and the two RNA species  (Gasior et al. 2020) for the free pool of protein is narrowly defined. In both models, the authors fixed two of the binding dynamics involved in protein-RNA complex formation to help ensure that the system would phase separate. Additionally, both papers fixed their protein and RNA diffusion coefficients to values based on the molecular weights of known protein and RNA species involved in phase separation in *Ashbya gossypii*. Thus, “competition” in both models is defined as the exchanging of protein molecules by the disassociation of one complex and the formation of the other. Although these simplifications help in understanding how protein-RNA interactions, coupled with competition for a shared resource, can define observable results, they ignore the potential influence of all protein-RNA interactions, as well as the impact of diffusion as like-complexes coalesce for droplet formation.

To fully explore these complex dynamics, a more methodical approach is required to quantify the role of different parameters. Given the multiple parameter dimensions to explore, it is difficult to determine which simplification to address first, leading us to consider sensitivity analysis for quantifying the differences between predictions as parameters are varied. Sensitivity analysis requires an input-output relationship, or a model  (Saltelli et al. 2008). The inputs are the parameter values and the output is an observable outcome of the model, referred to as the quantity of interest (QoI)  (Smith 2013). Sensitivity analysis is a useful tool when using computational and mathematical models to understand real world situations where the parameter values may be uncertain either from lack of data (epistemic) or inherent variations (aleatory)  (Smith 2013). These techniques can help scientists understand how outputs depend on the parameterization of the model, as well as quantify the uncertainty of the outputs  (Qian and Mahdi 2020). There is a tension between having many free parameters with wide ranges to explore, which is computationally expensive but allows for broader model behavior, and a restricted parameter regime. Thus, sensitivity analysis can help modelers understand the roles their modeling choices and parameters play in the situations their model describes. However, the informative power of sensitivity analysis is limited by the parameters, their ranges, and the methods that the modeler chooses  (Smith 2013; Qian and Mahdi 2020). This is often dictated by a priori estimates or computational restrictions  (Campolongo et al. 2011).

Sensitivity analyses can be classified as local or global depending on which parameters are allowed to vary. Local sensitivity analysis examines what happens when one parameter is varied at a time  (Jarrett et al. 2015). One of the simplest ways to measure the local sensitivity of a parameter is to estimate the derivative of the QoI with respect to the parameter of interest. However, it is known that this may result in an inaccurate assessment of the sensitivity since this substantially reduces the dimensionality that is being explored  (Saltelli and Annoni 2010). Global sensitivity analysis focuses on how model uncertainty can be attributed to uncertainty pertaining to a single parameter or a combination of several model parameters, while allowing all parameters to vary and is typically much preferred  (Campolongo et al. 2011). However, global methods require many more input samples, which can become problematic if each sample requires substantial computational time  (Saltelli et al. 2019).

Morris Method (MM) is a global sensitivity analysis technique that approximates local derivative-based methods but varies these over the global parameter regime to assess the variation in the derivative (local) index over the the global domain (Morris 1991). MM applies one at a time (OAT) methodology for understanding the influence of model parameters on outcomes of interest  (Campolongo et al. 2011; Qian and Mahdi 2020). To circumvent the local nature of OAT sampling and local derivative-based methodology, MM approximates the derivative using a finite difference scheme and then averages these many samples together to produce the statistics, such as the mean ($\mu ^*$) of the individual parameter on the QoI (Qian and Mahdi 2020). This mean is the individual effect of the parameter on the QoI while the other descriptive statistic produced by MM, the variance ($\sigma ^2$), pertains to the impact of the parameter due to nonlinearity and interactions with other parameters in the model  (Qian and Mahdi 2020). While both values can highlight the importance of a parameter and whether it is interacting with other parameters, there is limited information from MM. There is no consensus regarding the magnitude of $\mu ^*$ and $\sigma$ that determine significance. Instead, this is a qualitative judgement based on the scales, specific problem at hand and computational requirements (Qian and Mahdi 2020; Smith 2013). Thus, MM’s true value lies in grouping parameters based on their relative importance and reducing the parameter space for further exploration. Referred to as a ”screening” method, MM separates parameters into those that can be frozen without altering the QoI and those that have a higher impact (Qian and Mahdi 2020; Smith 2013).

Variance-based sensitivity techniques are global sensitivity analyses that apportion the amount of variance in the output from the model attributed to each parameter. These methods are quite popular due to the extensive information they can provide about the parameters used in the model. Further, these methods often do not have modeling constraints present in other techniques, such as linearity and monotonicity. One such technique is Sobol’ sensitivity analysis  (Saltelli et al. 2008). Sobol’ uses ANOVA-like decomposition to determine the impact of the parameters  (Jarrett et al. 2015). With this analysis, Sobol’ is able to compute the total impact, leading order effects, and higher order effects of each parameter on the model output. The total effects measures the relative variance in the quantity of interest that can be attributed to a single parameter alone or in conjunction with other parameter variations (e.g. individual and higher order effects). This provides a useful method of ranking or screening the parameters with respect to their importance. Higher order effects indicate interactions between parameters and how combined variations in parameters impacts the variance. Being able to determine these interactions can highlight complex and unknown relationships between the model dynamics that produce the phenomena we observe, a deficiency in other global techniques, such as MM. Unfortunately, variance-based methods are computationally expensive. The indices are defined via integrals whose dimensions scale with the number of parameters, so efficient methods are required for numerical estimation. Showing convergence of the estimates can be difficult, especially for larger models and parameter sets. These disadvantages suggest coupling screening methods with variance-based methods, as screening methods allow us to quickly identify the most important parameters, followed by variance-based methods to further study the parameter-output relationship.

In this work, we study the molecular dynamics and their cooperation underlying observable phenomena during intracellular liquid-liquid phase separation. Using the Cahn-Hilliard diffuse interface model with a Flory-Huggins free energy scheme put forth by Gasior et al. (2020) to describe two competing RNA species, we examine the impact of both the molecular diffusion coefficients and the binding dynamics on the time of phase separation and the fraction of $K_1$ droplets at the onset of separation. Here the fraction of $K_1$ droplets signifies the heterogeneity of the droplet field, as a fractional value of 0 or 1 would indicate a droplet field homogeneous in $K_2$ or $K_1$ droplets, respectively. Additionally, we vary the initial conditions from systems with equal quantities of both RNA species to systems skewed with increasingly more of one RNA species over the other to determine the density-dependence of the molecular interactions, as well as their impact on these LLPS attributes. Employing a methodology that combines sensitivity analyses, our work uses Morris Method screening to reduce the computationally expensive system with nine initial parameters to manageable systems with six or less free parameters. We then use Sobol’ sensitivity analysis to determine the influence of the parameters and how their interactions control both outputs under the different initial conditions. With this approach, we find that the protein-RNA binding dynamics impact both the time of phase separation and the composition of the droplet field at the onset of separation. For the time of phase separation, the impact of the mass action kinetics is derived from both the main effects of the individual parameters, as well as their interactions with each other. The fraction of $K_1$ droplets at the time of separation present more complicated results. Here, the diffusion coefficients of the protein-RNA complexes play a larger role in dictating the composition of the droplet field. Further, Sobol’ sensitivity analysis shows that this QoI is the result of interactions involving multiple (3+) parameters. Taken together, these results suggest that no single parameter is responsible for the observed droplet dynamics, especially those that can be attributed to a competitive environment for a shared resource. However, despite the complicated molecular dynamics revealed underlying these simple measurements, this methodical approach using combined sensitivity analyses establishes a framework for exploring droplet field dynamics in the future.

**Fig. 1:**
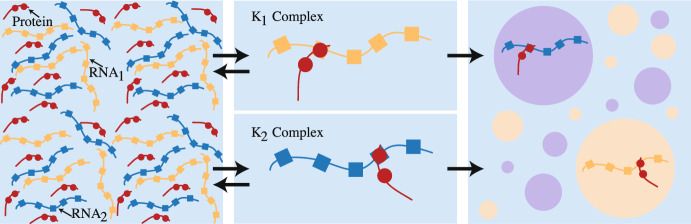
Schematic of two RNA species (yellow, blue) competing for the protein species (red). Monovalent interactions between the a protein molecule and an RNA molecule results in a protein-RNA complex ($K_1$ and $K_2$). These complexes then drive phase separation, creating two distinct droplet species that can coexist within the same droplet field (color figure online)

## Methods

### Mathematical Model of Intracellular Liquid-Liquid Phase Separation with RNA Competition

Here we describe the model presented in Gasior et al as it pertains to our analysis. Full discussion of the model can be found in  (Gasior et al. 2020). This model examines two RNA species competing for a common protein-binding partner via reversible monovalent interactions. As shown in Fig. 1, a single protein is capable of binding to an RNA molecule from RNA species 1 or 2 ($R_1$, $R_2$) to form a $K_1$ or $K_2$ complex, respectively, both of which drive phase separation. The model in equations (1)–(7) couples the Cahn-Hilliard, diffuse interface, phase field method, the Flory-Huggins free energy scheme, and reversible protein-RNA interactions to describe the binding and unbinding of protein and RNA to form complexes driving droplet formation. Equation (6) details the Flory-Huggins free energy scheme, where $A = 0.2$ is a scaling factor and each $\chi$ is an interaction parameter  (Weber et al. 2019) describing the pairwise de-mixing interactions between each of the protein-RNA complexes ($\chi _{K_1K_2}$) and the protein-RNA complexes with the solvent ($\chi _{K_1S}$, $\chi _{K_2S}$). For simplicity, we assume $\chi _{K_1S}=\chi _{K_2S}=\chi _S$. All parameter values are detailed in Table 1. Additionally, in Gasior et al., $0<M(K_1,K_2)\le 1$ is a mobility function that scales linearly in a phase-dependent manner, so as to mimic the loss in component mobility within the droplet due to the highly viscous environment. However, previous work by Gasior and Zhao et al. showed the minimal role of phase-dependent diffusion prior to separation so, for simplicity, we set $M(K_1,K_2)=1$.

**Table 1:** Model parameter values and sensitivity analysis sampling ranges

Parameter	Definition	Reported	Sensitivity analysis	Units
		value	range	
$\lambda _P$	Diffusion rate of free protein	$37.5 \times 10^{-1}$	[0, 5]	$\upmu$m$^2/$s
$\lambda _{R_1}$	Diffusion rate of free RNA1	$5.8 \times 10^{-1}$	[0, 2]	$\upmu$m$^2/$s
$\lambda _{R_2}$	Diffusion rate of free RNA2	$1.45 \times 10^{-1}$	[0, 2]	$\upmu$m$^2/$s
$\lambda _{K_1}$	Diffusion rate of the $K_1$ complex	$5 \times 10^{-1}$	[0, 2]	$\upmu$m$^2/$s
$\lambda _{K_2}$	Diffusion rate of the $K_2$ complex	$1.39 \times 10^{-1}$	[0, 2]	$\mu$m$^2/$s
$a_1$	Association rate of $K_1$	$1.0 \times 10^0$	[0.5, 1.5]	1/s
$a_2$	Disassociation rate of $K_1$	$1.0 \times 10^{-2}$	[0, 0.5]	1/s
$a_3$	Association rate of $K_2$	$1.0 \times 10^0$	[0.5, 1.5]	1/s
$a_4$	Disassociation rate of $K_2$	$1.0 \times 10^{-2}$	[0, 0.5]	1/s
$\chi _S$	Demixing energy between protein-	4.25	${-}$	${-}$
	RNA complexes and solvent			
$\chi _{K_1K_2}$	Demixing energy between different	4.5	${-}$	${-}$
	protein-RNA complexes			

**Fig. 2:**
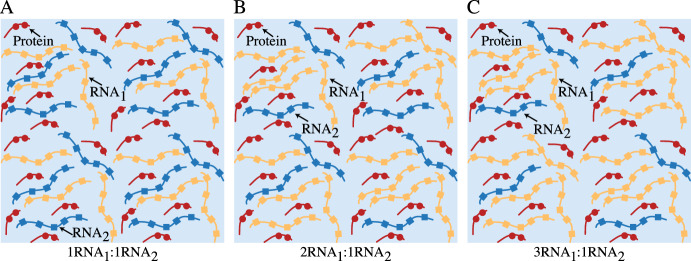
Three different initial ratios of RNA in a well-mixed system are explored. Gasior et al. developed their model using equal amounts of $R_1$ and $R_2$, or $1R_1:1R_2$ (**A**). We also examine systems beginning with $2R_1:1R_2$ (**B**) and $3R_1:1R_2$ (**C**). For all three systems, the initial amount of free protein remains the same and is equal to the sum of the two RNA fractions ($P \approx R_1+R_2$) (color figure online)

In this model, it is assumed there are no preformed protein-RNA complexes $(K_1(x, y, 0) = K_2(x, y, 0) = 0)$. Gasior et al. used a random uniform initial distribution of protein and the initial amount of each RNA species across the entire domain was calculated to be half of the protein $(R_1 = R_2 =\frac{1-P}{2})$. Further, it was assumed that the total amount of protein and RNA are conserved, resulting in a system wherein $K_1$ and $K_2$ formation is limited by the initial conditions. Here, these conditions are referred to as $1R_1:1R_2$ initial conditions. We also consider systems with initial condition ratios of $2R_1:1R_2$, where $R_1 = 2 \times R_2$, as well as $3R_1:1R_2$ with $R_1 = 3 \times R_2$, as shown in Fig. 2. In all three cases, $R_1 +R_2 \approx P$.

1 $$\begin{aligned} \frac{\partial K_1}{\partial t}&= \nabla \cdot \Big [\lambda _{K_1}M(K_1,K_2)\nabla \Big (\frac{\delta F}{\delta K_1} \Big ) \Big ]+a_1PR_1-a_2K_1 \end{aligned}$$

2 $$\begin{aligned} \frac{\partial K_2}{\partial t}&= \nabla \cdot [\lambda _{K_2}M(K_1,K_2)\nabla \Big (\frac{\delta F}{\delta K_2}\Big )]+a_3PR_2-a_4K_2 \end{aligned}$$

3 $$\begin{aligned} \frac{\partial P}{\partial t}&= \nabla \cdot \Big [\lambda _P M(K_1,K_2) \nabla P \Big ] - a_1PR_1+a_2K_1-a_3PR_2+a_4K_2 \end{aligned}$$

4 $$\begin{aligned} \frac{\partial R_1}{\partial t}&= \nabla \cdot \Big [\lambda _{R_1} M(K_1,K_2) \nabla R_1 \Big ] - a_1PR_1 + a_2K_1 \end{aligned}$$

5 $$\begin{aligned} \frac{\partial R_2}{\partial t}&= \nabla \cdot \Big [\lambda _{R_2} M(K_1,K_2) \nabla R_2 \Big ] - a_3PR_2 + a_4K_2 \end{aligned}$$

6 $$\begin{aligned} F&= \int _\Omega \Big ( \frac{\epsilon ^2}{2} [|\nabla K_1|^2+|\nabla K_2|^2+A \cdot (K_1 \ln K_1 + K_2 \ln K_2 + S \ln S \nonumber \\&+ \chi _{K_1 K_2}K_1 K_2 + \chi _{K_1S}K_1S + \chi _{K_2S}K_2S)dx \end{aligned}$$

7 $$\begin{aligned}&K_1 +K_2 + S = 1 \end{aligned}$$

With this model established, Gasior et al. explored RNA competition for a shared protein-binding partner by altering the rate at which the $K_1$ complex disassociates ($a_2$) and the rate at which the $K_2$ complex associates ($a_3$). By varying these two values, the free protein was released back into the general solution from the $K_1$ complex and was available to interact with $R_2$ to form the $K_2$ complex. The authors set $a_1 = 1.0$ and $a_4 = 0.01$ to increase the probability that the system would phase separate and then varied $a_2$ and $a_3$ such that $a_2 \in (0,1.1]$ and $a_3 \in (0,1.1]$. The authors found that in a competitive RNA environment, there were four general outcomes for the droplet field composition at the onset of separation: a homogeneous $K_1$ droplet system, a homogeneous $K_2$ droplet system, a heterogeneous system where both $K_1$ and $K_2$ droplets are present at the onset of phase separation, or a system that does not phase separate at all. Lack of phase separation was confined to systems where $a_2$ was too high and $a_3$ was too low, indicating that the $K_1$ complex was unstable and releasing free protein too quickly, but the $K_2$ complex was too slow-to-form to sufficiently accumulate for driving phase separation.

**Fig. 3:**
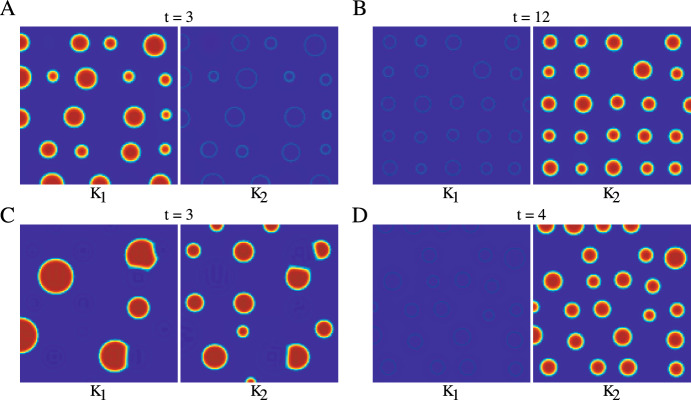
Four examples of the binding dynamics explored by Gasior et al. where the disassociation rate of $K_1$ ($a_2$) and the association rate of $K_2$ ($a_3$) are varied. Dynamics presented here are captured at the time of separation. **A** A fast-forming and stable $K_1$ complex and a slow-forming and stable $K_2$ complex ($(a_2,a_3) = (0.01,0.25)$) results in quickly phase separating system ($t = 3$s) homogeneous in $K_1$ droplets. **B** A fast-forming but weak $K_1$ complex and a slow-forming but stable $K_2$ complex ($(a_2,a_3) = (1.0,0.25)$) results in a $K_2$ dominated droplet field at the onset of separation. This system takes the longest to phase separate at $t = 12$s. **C** Two quickly forming and stable protein-RNA complexes ($(a_2,a_3) = (0.01,1.0)$) resulting a quickly phase separating ($t = 3$s) and heterogeneous droplet field. **D** A quickly forming but weak $K_1$ complex and a quickly forming and stable $K_2$ complex ($(a_2,a_3) = (1.0,1.0)$) that quickly produces a droplet field homogeneous in $K_2$ droplets ($t = 4$s) (color figure online)

Figure 3 shows the influence of these two parameters on initial droplet field composition and the time of phase separation in four representative systems. In these examples, $M(K_1,K_2) =1$. If a quickly forming and stable $K_1$ complex is created along with a slow forming and stable $K_2$ complex, it is $K_1$ that will drive phase separation and comprise the entire droplet field at the onset of separation, as shown in Fig. 3A. But if the $K_1$ complex is not stable, the $K_2$ complex will drive phase separation and dominate the droplet field at this time point, even if it is a complex that is slow to form, as shown in Fig. 3B, D. Further, it is the systems where the $K_1$ complex is unstable and the $K_2$ complex is slow-to-form that take the longest to phase separate (Fig. 3B). Finally, if both complexes are quick to form and stable, we observe heterogeneity at the onset of phase separation (Fig. 3C). Here both $R_1$ and $R_2$ are abundant at the start of the simulation and have mass-action kinetics that promote the formation of stable $K_1$ and $K_2$ complexes. This competitive scenario allows for mutual coexistence of the two droplet species at the onset of separation. We now describe the screening methods used to group the parameters based on the outputs suggested by this discussion.

### Morris Method Screening

**Fig. 4:**
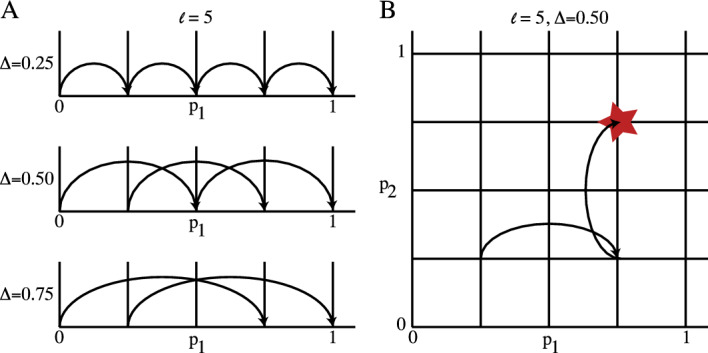
Morris Method schematic for parameter space sampling for a single parameter (**A**) and multiple parameters (**B**). **A** The parameter space for a single parameter ($p_1$) is divided into five levels $l = 5$. The space can then be sampled using a potential step size of $\Delta = 0.25$, $\Delta = 0.50$, or $\Delta = 0.75$. **B** The multi-parameter space ($p_1$, $p_2$) is divided up into five levels per parameter ($l = 5$) and the sampled using a step size of $\Delta = 0.5$ for both $p_1$ and $p_2$. Ultimately, these representative examples illustrate the parameter space exploration possible under large, varying $\Delta$ values. In this work, $\Delta = 0.001$

Morris Method screening (MM) approximates the local sensitivity (e.g. the derivative of the output with respect to each other) and develop statistics of this measure over the entire parameter space. The estimates also depend on assumptions regarding the parameter distributions. While other methods restrict the distributions with respect to independence (e.g. Sobol’), MM is very general - even though the estimates obtained depend on the assumed parameter distributions. For consistency between MM and Sobol’, we assume each parameters are independent and identically distributed (iid). The domain of each parameter $X_j$ is scaled to a range from [0,1] and then divided into *l* levels. A schematic for sampling a single parameter space and multiple parameter spaces is shown in Fig. 4. The jth elementary effect (e.g. the approximate derivative of *Y* in the $X_j$ parameter direction) is,

8 $$\begin{aligned} EE_j = \frac{Y(X_1, \cdot \cdot \cdot , X_j + \Delta , \cdot \cdot \cdot , X_9) - Y(X_1, \cdot \cdot \cdot , X_9)}{\Delta } \end{aligned}$$

where the value of $\Delta$ is chosen from $\Delta \in \{ \frac{1}{l-1},1-\frac{1}{l-1}\}$  (Saltelli et al. 2008; Smith 2013; Qian and Mahdi 2020; Campolongo et al. 2011, 2007). This process is carried out for a total of r runs. Each $(X_1, \cdot \cdot \cdot , X_9)$ is a random sample from the chosen parameter space and taken to be independent and identically distributed for the r runs. The elementary effects are then averaged to generate the sensitivity measures $\mu _j^*$ and $\sigma _j^2$:

9 $$\begin{aligned} \mu _j^*&= \frac{1}{r} \sum _{i=1}^r |EE_j^i| \end{aligned}$$

10 $$\begin{aligned} \sigma _j^2&= \frac{1}{r-1} \sum _{i=1}^r (EE_j^i - \mu _j^*)^2 \end{aligned}$$

The higher the value of $\mu _j^*$, the more influential the parameter $X_j$ is on the outcome. Further, a higher value of $\sigma _j^2$ indicates nonlinearity and potential interaction with other parameters of the model  (Saltelli et al. 2008; Smith 2013; Qian and Mahdi 2020).

MM was carried out on the four binding dynamics $(a_1, a_2, a_3, a_4)$ and five diffusion coefficients $(\lambda _P, \lambda _{R_1}, \lambda _{R_2}, \lambda _{K_1}, \lambda _{K_2})$, thus $j=1,\cdot \cdot \cdot ,9$ for all three initial conditions ($1R_1:1R_2$, $2R_1:1R_2$, $3R_1:1R_2$). The ranges used for these nine parameters, prior to being scaled to a [0, 1] range are listed in Table 1. These parameter ranges were chosen based on previous sampling of the parameter space performed by Gasior et al., as well as to encourage phase separation of the system. Parameter regimes with small $a_1$,$a_3$ values or large $a_2$,$a_4$ values could prevent phase separation of the system. Further the step-size was set such that $\Delta = 0.001$ and the total number of runs for calculating $\mu _j^*$ and $\sigma _j^2$ was $r=1000$. Code from  Herman and Usher (2017) was used  (Iwanaga et al. 2022).

For each simulation, data was recorded every $t = 1$ seconds, meaning that there is no distinction between systems with a fractional time of separation ($\lceil t \rceil$). If a simulation did not separate by $t = 1000$ seconds, a value of $t = 1000$s was assigned as the time of separation and the fraction of $K_1$ droplets at the ”onset of separation” in these well-mixed systems was set as a false fraction of 1.25 so as to distinguish them from systems that were dominated by either $K_1$ or $K_2$. For all initial conditions, these well-mixed systems at $t = 1000$ seconds accounted for less than $~20\%$ of all simulations, as shown in Table 2 of the Appendix. All data are available upon request.

### Sobol’ Sensitivity Analysis

Sobol is a variance-based method that uses ANOVA decomposition to calculate the effects of each parameter on the variance of the given output  (Sobol 2001; Jarrett et al. 2017). The development of Sobol’ indices rests on the Sobol’ decomposition (Chastaing et al. 2012). As long as the parameters are iid, one can decompose the model output in a unique orthogonal way using conditional probabilities. However, it should be noted that there are formulations of Sobol’-like sensitivities that do not require independence (Da Veiga et al. 2021; Chastaing et al. 2015; Borgonovo et al. 2012); however, for the purposes of this paper we assume iid. Several quantities of interest include the primary influence of a parameter on the output variance ($S_1$), the total impact of each parameter on the output variance ($S_T$), and all interactions between the parameter and other parameters of interest ($S_T - S_1$). The primary impact, or main effects, of a parameter ($S_1$) is defined as:

11 $$\begin{aligned} S_{1i} = \frac{V_{X_i}(E_{X_{\sim i}}(Y|X_i))}{V(Y)} \end{aligned}$$

and the total impact of the parameter ($S_{Ti}$) is defined as:

12 $$\begin{aligned} S_{Ti} = \frac{E_{X_{\sim i}}(V_{X_i}(Y|X_{\sim i}))}{V(Y)} \end{aligned}$$

where Y is the output, $X_i$ is the parameter of interest, $X_{\sim i}$ are all other parameters excluding the parameter of interest, V is the variance, and E is the expected value  (Sobol 2001; Saltelli et al. 2010; Smith 2013). While Sobol can calculate all interactions between parameters, including interactions between multiple parameters, one value of paraticular interest is $S_2$, or the one-one interaction between the given parameter $X_i$ and another parameter of interest, $X_{\sim i}$, defined by considering the incremental variances. One can understand $S_{1i}$ as the ratio of the variance of the expected value, given knowledge of parameter $X_i$ to the total variance. We can then understand the second order effects as the ratio of the variance of the expected value given knowledge of $X_i$ and $X_j$ in all combinations, to the total variance:

13 $$\begin{aligned} S_{2_{ij}} = \frac{V_{X_{ij}}(E_{X_{\sim ij}}(Y|X_i,X_j))-V_i-V_j)}{V(Y)} \end{aligned}$$

Code from  Herman and Usher (2017) was used to calculate all indices. In this algorithm, the number of evaluations or runs of our simulation (*k*) necessary to compute $S_T$, $S_1$, and $S_2$ for each outcome is:

14 $$\begin{aligned} k = n \cdot (i + 2) \end{aligned}$$

where *n* is the number of Sobol’ samples and *i* is the number of parameters varied.

Global sensitivity indices are typically estimated using sampling from joint distributions  (Renardy et al. 2021). The choice of the underlying distributions can have profound effects on the estimates  (Plischke et al. 2013; Kucherenko et al. 2012; Borgonovo et al. 2016). Uniform or normal distribution are typical choices. However, if there is deeper knowledge of the specific parameters other distributions can be used  (Blower and Dowlatabadi 1994). In this study, we do not have detailed knowledge of the biophysical statistics and therefore chose a uniform distribution. We acknowledge that there could be hidden biophysical interactions between parameters in the crowded geometry of the cellular environment, where protein and RNA interactions between certain binding sites could produce changes in folding that subsequently impact the diffusivity of the resulting complex. However, the mathematical model of Gasior et al. (2020) addresses this issue by assuming parameter independence in their structure; the species created by the protein-RNA interactions ($K_1,K_2$) have their own properties and diffusivities that are based on their molecular weight, independent of the rate at which these complexes associate or disassociate. Similarly, here, we assume the processes are uncorrelated.

To determine the indices for each parameter, the number of evaluations was doubled until $(S_{Ti_{2k}}-S_{Ti_k})/S_{Ti_k} < 0.1$. Less than $10\%$ change in $S_T$ would indicate these statistics had stopped oscillating and had converged to their true values. Convergence is always an issue for Sobol’ and this method ensures that there are enough samples to conclude that our estimates have converged. For each simulation, data was recorded every $t = 1$ seconds, meaning that there is no distinction between systems with a fractional time of separation ($\lceil t \rceil$). If a simulation did not separate by $t = 10$s, a value of $t = 10$s was assigned as the time of separation and the fraction of $K_1$ droplets at the "onset of separation" in these well-mixed systems was set as a false value of 1.25 so as to distinguish them from systems that were dominated by either $K_1$ or $K_2$. For the final set of evaluations necessary for convergence under all three initial conditions, $< 10\%$ took longer than $t = 10$s to phase separate, as shown in Table 2 of the Appendix. Further convergence results are provided in Table 3 of the Appendix. All data are available upon request.

**Fig. 5:**
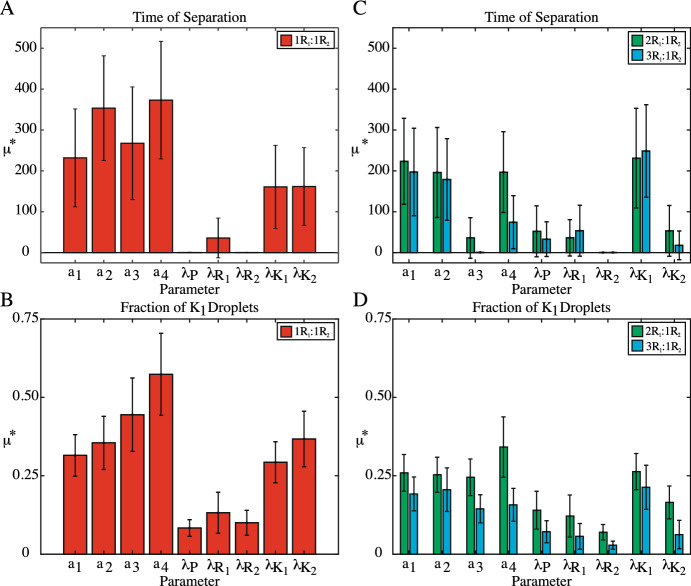
Morris Method screening for the impact of the four binding dynamics ($a_1--a_4$) and the five diffusion coefficients ($\lambda _P$, $\lambda _{R_1}$, $\lambda _{R_2}$, $\lambda _{K_1}$, $\lambda _{K_2}$) on the time of phase separation (**A** & **C**) and the fraction of $K_1$ droplets at the onset of phase separation (**B** & **D**). The $\mu ^*$ value is shown in color for each set of initial conditions, with error bars indicating $\sigma$. Initial conditions with a $1R_1:1R_2$ ratio are shown in **A** & **B** and skewed initial conditions are shown in **C** & **D**. **A** & **B** MM screening shows that six of the original nine parameters impact both outcomes: the four binding dynamics and the diffusion coefficients for the competing protein-RNA complexes ($\lambda _{K_1}$, $\lambda _{K_2}$). Skewing the initial conditions in favor of $R_1$ further reduces the parameter set. **C** For the time of phase separation, with $2R_1:1R_2$ initial conditions, MM screening now highlights four influential parameters: the $K_1$ binding dynamics ($a_1$, $a_2$), the $K_2$ disassociation rate ($a_4$), and the $K_1$ diffusion coefficient ($\lambda _{K_1}$). For the $3R_1:1R_2$ initial conditions, the parameter set is reduced to three parameters: the $K_1$ binding dynamics ($a_1$, $a_2$) and the $K_1$ diffusion coefficient ($\lambda _{K_1}$). **D** For the $2R_1:1R_2$ initial conditions, there are now five parameters impacting the fraction of $K_1$ droplets: the four binding dynamics and the diffusion coefficient for the $K_1$ complex ($\lambda _{K_1}$). Skewing the initial conditions further towards $R_1$ with a $3R_1:1R_2$ ratio reduces the parameter set to three parameters: the binding dynamics and the diffusion coefficient for the $K_1$ complex ($a_1$,$a_2$, $\lambda _{K_1}$)

## Results

### Morris Method Screening Reduces the Varied Parameter Set to Dynamics Involved in Droplet Formation

To identify the most important molecular dynamics contributing to the time of phase separation and the droplet field composition at this time point, Morris Method (MM) screening was carried out. Figure 5 shows the MM screening results for all four binding dynamics ($a_1-a_4$) and diffusion coefficients ($\lambda _P$, $\lambda _{R_1}$, $\lambda _{R_2}$, $\lambda _{K_1}$, $\lambda _{R_2}$) in simulations with $1R_1:1R_2$ initial conditions (Fig. 5A, B), as well as initial conditions skewed towards $K_1$ formation (Fig. 5C and D). In each plot, the colored bars are the $\mu ^*$ values for each parameter, while the associated error bars shown are the standard deviations ($\sigma$).

For systems with $1R_1:1R_2$ initial conditions in Fig. 5A and B, the same subset of six parameters controls both the time of separation and droplet field composition at phase separation. Despite differences in overall scale, the discrepancy between parameters considered important by MM screening for each output and those that will not be included in Sobol’ analysis, is similar. In Fig. 5A, the $\mu ^*$ values for the binding dynamics ($a_1-a_4$) and the protein-RNA complex diffusion coefficients($\lambda _{K_1}$, $\lambda _{K_2}$) are at least $\mu ^* = 1.61 \times 10^2$, while the diffusion coefficients for the free components ($\lambda _P$, $\lambda _{R_1}$, $\lambda _{R_2}$) have a maximum $\mu ^* = 3.59 \times 10^1$. In Fig. 5B, the binding dynamics and protein-RNA complex diffusion coefficients have a minimum $\mu ^* = 2.93 \times 10^{-1}$, while the diffusion of the free components have a maximum value of $\mu ^* = 1.32 \times 10^{-1}$. Thus, for systems with $1R_1:1R_2$ initial conditions, the timescale on which phase separation is observed, as well as the composition of the droplet field, is due to the strength of the interactions between the protein and RNAs, as well as how quickly the protein-RNA complexes can find each other to form droplets.

Skewing the initial conditions to favor the $K_1$ complex formation shifts the impact of the molecular dynamics on both the time of separation and the fraction of $K_1$ droplets at the onset. Figure 5C, D show the results of MM screening when applied to systems with an initial ratio of $2R_1:1R_2$ (green) and $3R_1:1R_2$ (blue). For the time of phase separation in Fig. 5C, skewed initial conditions decreases the impact of $K_2$-associated parameters. With $2R_1:1R_2$ initial conditions, there is now a subset of four important parameters influencing the time of phase separation: $a_1$, $a_2$, $a_4$, and $\lambda _{K_1}$. Here, the $K_2$ disassociation rate ($a_4$) is the only $K_2$-associated parameter deemed important under these skewed initial conditions. Like the behavior in Fig. 5A, there is a distinct separation between these four parameters and those that do not impact the time of phase separation, with the smallest $\mu ^*$ value for this four-parameter subset as $\mu ^* = 1.96 \times 10^2$, but the largest $\mu ^*$ for the other five parameters being $\mu ^* = 5.32 \times 10^1$. Skewing further towards $K_1$ formation with the $3R_1:1R_2$ initial conditions further reduces the parameter set. Now, the most important parameters influencing the time of phase separation are those specifically associated with $K_1$ complex formation ($a_1$, $a_2$) and droplet coalescence ($\lambda _{K_1}$).

Favoring $K_1$ complex formation via the initial conditions also reduces the number of dynamics impacting the fraction of $K_1$ droplets at the onset of phase separation, as shown in Fig. 5D. Like the $1R_1:1R_2$ initial conditions, the diffusion coefficients for the free protein and RNA species are not as important as the other parameters in determining the composition of the droplet field at the onset of separation. Further, skewing the initial conditions towards $K_1$ formation reduces the importance of $K_2$-associated parameters, such as the $K_2$ diffusion coefficient ($\lambda _{K_2}$) with the $2R_1:1R_2$ initial conditions and the $K_2$ binding dynamics and diffusion coefficient ($a_3-a_4$, $\lambda _{K_2}$) for the $3R_1:1R_2$ initial conditions. Thus, by using our initial conditions to favor the formation of the $K_1$ complex, we have reduced our parameter set to five parameters ($a_1-a_4$, $\lambda _{K_1}$) for the $2R_1:1R_2$ initial conditions and to three parameters ($a_1$, $a_2$, $\lambda _{K_1}$) for the $3R_1:1R_2$ initial conditions. However, unlike the results in Fig. 5A–C, the distinction between important dynamics impacting the droplet field composition and the rest of the parameter set is less distinct with initial conditions favoring $K_1$ formation, as indicated by $\mu ^* < 0.25$ for all nine parameters under the $3R_1:1R_2$ initial conditions.

Ultimately, while Morris method is useful for distinguishing between important and unimportant parameters, it is not designed for parameter ranking. Thus, comparing two parameters and comparing how variations impact the QoIs requires a different sensitivity method. Sobol’ sensitivity analysis is one of the most flexible and well-studied methods for sensitivity ranking. In the next sections, we exploit our results from Morris Method to reduce the parameter space Sobol’ sensitivity analysis, making it computationally feasible.

**Fig. 6:**
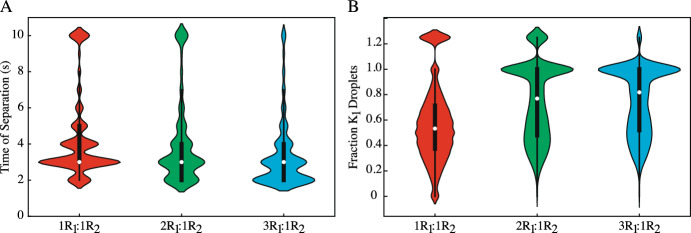
Violin plots of the Sobol’ sampling results for **A** the time of phase separation and **B** fraction of $K_1$ droplets at the onset of separation. Systems with a time of separation longer than 10 seconds were set to $t = 10$s and the fraction of $K_1$ droplets was set to a false value of 1.25, resulting in the clusters at these values in all plots in (**A**) and (**B**). For all data, a box plot (black) is superimposed over the density distribution of the data (color) and the median of each data set is indicated with a white dot. **A** Skewing the initial conditions towards $K_1$ formation forces the systems to separate faster e.g. the distribution shifts down. Shifting the initial conditions from $1R_1:1R_2$ to $3R_1:1R_2$ results in an overall reduction in the time of phase separation, with a smaller subset of the data taking longer than $t = 10$s to separate and the bulk of the data distribution shifts from $t =3$s to a $t=2$s time of separation. However, this does not change the median of the data sets, which remains at $t=3$s for all initial conditions. **B** For the fraction of $K_1$ droplets at the onset of phase separation, skewing the initial conditions shifts the data density and median to larger fractions of $K_1$ droplets (e.g. the distributions shift up). For the $1R_1:1R_2$ initial conditions, the data presents a roughly normal distribution, with a median of 0.533. But, for the $3R_1:1R_2$ initial conditions, the median is 0.818

### Sobol’ Sampling Distributions Highlight the Importance of the Initial Conditions on QoIs

While Morris Method screening can only highlight the importance of parameters involved in producing the observable droplet field dynamics, Sobol’ analysis allows for a more in-depth understanding of how parameters impact these outcomes. Upon completion of Morris Method screening, the parameter set was reduced from nine total varied parameters to six ($1R_1:1R_2$ for time and fraction of $K_1$ droplets), five ($2R_1:1R_2$ for fraction of $K_1$ droplets), four ($2R_1:1R_2$ for time), and three ($3R_1:1R_2$ for time and fraction of $K_1$ droplets) varied parameters. The parameter spaces of these reduced subsets were then sampled while all other parameters were held at the values outlined in Table 1, as established by Gasior et al.

To understand the influence and the limitations of the skewed initial conditions on the time of separation and the composition of the droplet field, violin plots are shown in Fig. 6. In both Fig. 6A, B, a box plot (black) that captures quartiles 1-3 is superimposed over the density distribution of the data (color). The median of each data set is indicated with a white dot. These violin plots capture the data of the final set of evaluations (simulation runs) for each output under the three different initial conditions. The number of evaluations in each plot can be found in Table 3 in the Appendix.

Figure 6 shows the distribution of the Sobol’ data under all three initial conditions. For the time of phase separation in a $1R_1:1R_2$ system (Fig. 6A), most simulations phase separated at $t = 3$ seconds, with a median at $t = 3$s and a 2 second interquartile range ($Q_1 = 3$s, $Q_3 = 5$s). Further, at the time of phase separation, the compositions of these droplet fields are varied and heterogeneous, with the data displaying a semi-normal distribution for the fraction of $K_1$ droplets present (Fig. 6B). Here, the median fraction of $K_1$ droplets is 0.533 with an interquartile range of 0.339 ($Q_1 = 0.375$, $Q_3 = 0.714$). Additionally, 9.76% of the systems took longer than 10 seconds to phase separate, thus accounting for the larger data cluster at $t = 10$ seconds in Fig. 6A, and the false fraction of $K_1$ droplets set to 1.25 in Fig. 6B.

**Fig. 7:**
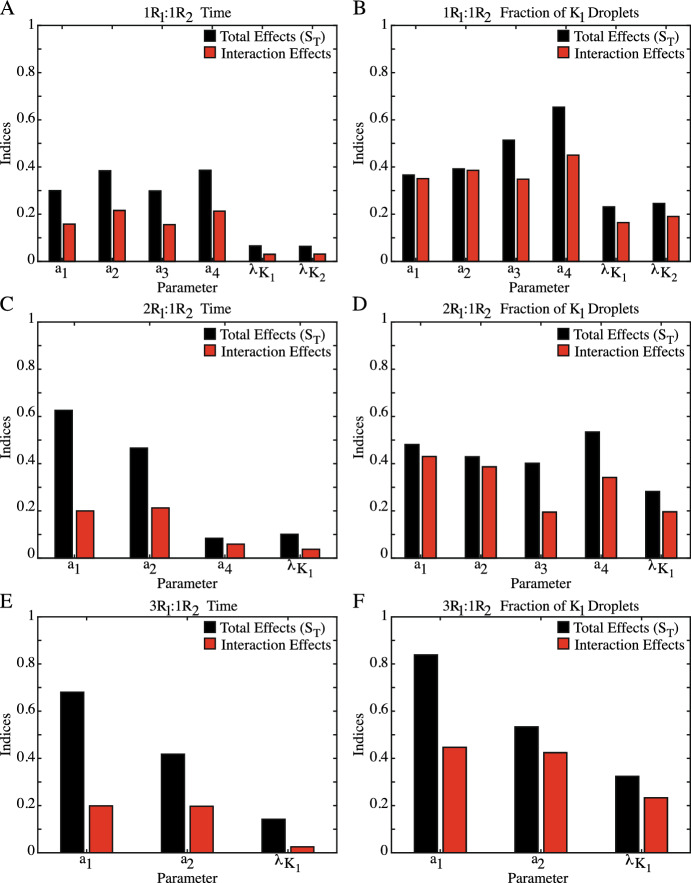
The results of Sobol’ sensitivity analysis for both the time of separation (**A**, **C**, **E**) and the fraction of $K_1$ droplets at the onset of separation (**B**, **D**, **F**). The total effects of each parameter ($S_T$) are shown in black, while the interaction effects ($S_T-S_1$) are in red. **A** The time of separation in systems with $1R_1:1R_2$ is impacted by the binding dynamics of the $K_1$ and $K_2$ complexes ($a_1-a_4$). Of these total effects, the interactions of the binding dynamics with other parameters comprise roughly half of the total impact ($52-56\%$). **C**, **E** For $a_1$ and $a_2$ under both the $2R_1:1R_2$ (**C**) and $3R_1:1R_2$ (**E**) initial conditions, the interaction effects account for less than half of each parameter’s impact on the time of separation. **B**, **D**, **F** While the diffusion coefficients exert more influence over the fraction of $K_1$ droplets at separation, the binding dynamics still impact this output most, as shown by the total effects, with the interaction effects comprising at least half ($\ge 48\%$) of the total effects for all parameters under all initial conditions (color figure online)

Skewing the initial conditions towards $K_1$ formation shifts the droplet field composition but has a lesser impact on the time of phase separation. While these biased initial conditions reduce the number of systems that take longer than $t = 10$ seconds to phase separate and shifts the data distribution to $t = 2$s for the $3R_1:1R_2$ initial conditions, the median time of phase separation remains at $t = 3$ seconds with an interquartile range of 2 seconds ($Q_1 = 2$s, $Q_3 = 4$s). However, these small changes in the time of phase separation are accompanied by a dramatic shift in the droplet field composition. Now, most of the droplet fields are $K_1$-homogeneous in $K_1$ droplets, with a median $K_1$ fraction of 0.818 for the $3R_1:1R_2$ initial conditions. Thus, in systems favoring the formation of the $K_1$ complex, mostly homogeneous droplet fields struggle to force phase separation any faster than the already quick timescale present under more equal initial conditions. Rather, this timescale may be more dependent upon the parameters. To understand what molecular interactions produce these dynamics, Sobol’ analysis is employed in the next section.

### Sobol’ Analysis Reveals Complex, Multi-Parameter (3+) Interactions Underlying QoIs

**Fig. 8:**
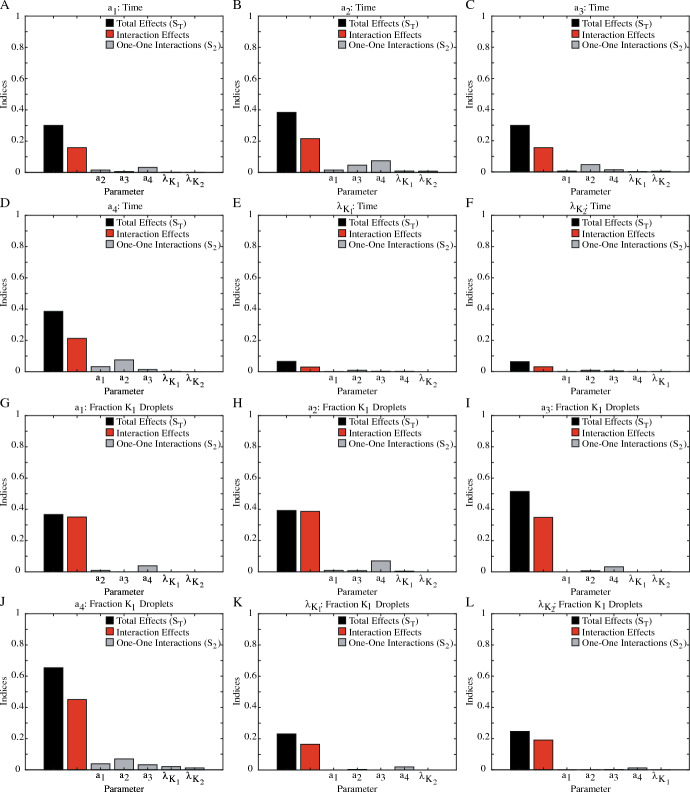
Sobol’ sensitivity analysis of parameter impact on the time of phase separation and the fraction of $K_1$ droplets at separation onset under $1R_1:1R_2$ initial conditions. The total effects ($S_T$, black) and total interaction effects ($S_T-S_1$, red) from Fig. 7A and B are again shown here. Additionally, the impact of the one-one interaction effects for each parameter ($S_2$) are shown in gray. Note that for some parameters, numerical calculations by  (Herman and Usher 2017) resulted in $-0.01<S_2<0$. These values were rounded to $S_2=0$. For the time of phase separation (A-F), the impacts of the one-one interactions are small. When summed, these one-one interactions comprise roughly half ($34-72\%$) of the interaction effects. For the composition of the droplet field at the time of separation (G-L), the one-one interactions of parameters carry little influence, with the sum of the gray bars for each parameters $\le 38\%$ of the total interaction effects (color figure online)

**Fig. 9:**
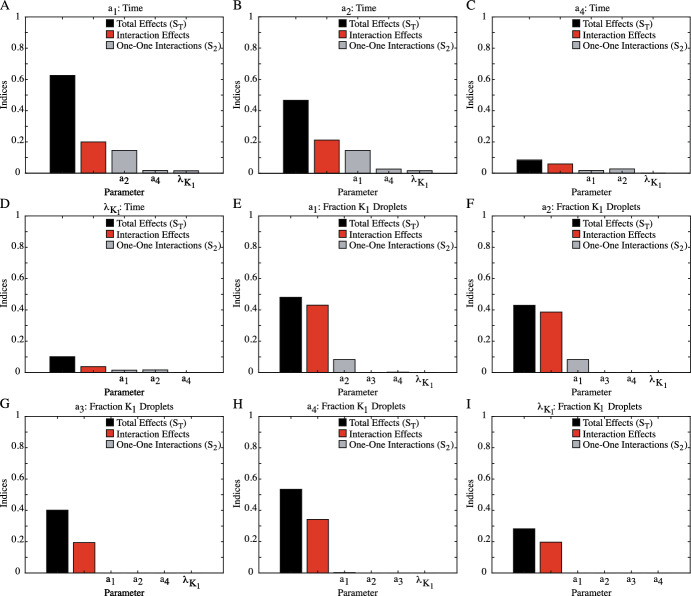
Sobol’ sensitivity analysis of parameter impact on the time of separation (**A**–**D**) and the fraction of $K_1$ droplets at separation (**E**–**I**) under $2R_1:1R_2$ initial conditions. The total effects ($S_T$, black) and total interaction effects ($S_T-S_1$, red) from Fig. 7C, D are again shown here. Additionally, the impact of the one-one interaction effects for each parameter ($S_2$) are shown in gray. Note that for some parameters, numerical calculations by  (Herman and Usher 2017) resulted in $-0.05<S_2<0$. These values were rounded to $S_2=0$. **A**–**D** With the skewed initial conditions, the binding dynamics controlling $K_1$ formation are the most impactful on the time of separation. The total interaction effects of both $a_1$ and $a_2$ (red) are almost equal to the one-one interactions with each other, as shown in **A** & **B**. **C** & **D** While impact of $a_4$ and $\lambda _{K_1}$ is less, both parameters primarily interact with the $a_1$ and $a_2$ parameters as well. **E**–**I** For the composition of the droplet field at separation, the one-one interactions between parameters are much less than the total interaction effects. Thus, it is the interactions between 3+ parameters, not one-one interactions, that control the observed droplet field composition (color figure online)

**Fig. 10:**
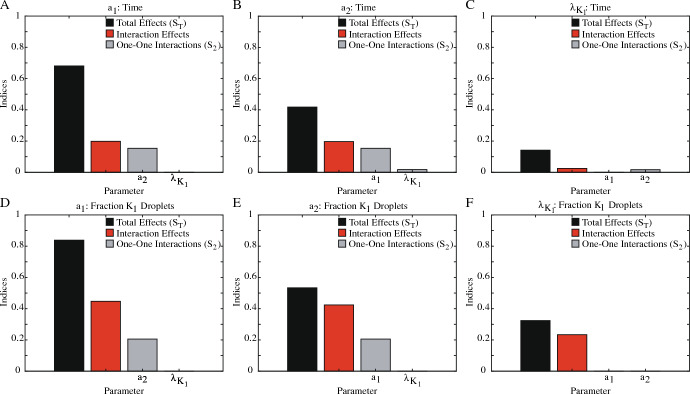
Sobol’ sensitivity analysis of parameter impact on the time of separation (**A**–**C**) and the fraction of $K_1$ droplets at the onset of separation (**D**–**F**) under $3R_1:1R_2$ initial conditions. The total effects ($S_T$, black) and the total interaction effects ($S_T-S_1$, red) from Fig. 7 are again shown here. Additionally, the impact of the one-one interaction effects for each parameter ($S_2$) are shown in gray. Note that for some parameters, numerical calculations by  (Herman and Usher 2017) resulted in $-0.01<S_2<0$. These values were rounded to $S_2=0$.Note that for some parameters, numerical calculations by  (Herman and Usher 2017) resulted in $-0.06<S_2<0$. These values were rounded to $S_2=0$. (A-C) With the skewed initial conditions, the binding dynamics controlling $K_1$ formation are the most impactful on the time of phase separation. The total interaction effects for both $a_1$ and $a_2$ (red) are almost equal to the one-one interactions with each other, as shown in **A** & **B**. **C** For the $K_1$ diffusion coefficient ($\lambda _{K_1}$) , the total impact due to interaction effects is primarily composed of one-one interactions with $a_2$, the disassociation rate of the $K_1$ complex. For the composition of the droplet field at the onset of separation (**D**–**F**), the one-one interactions between all parameters are less than half of the total interaction effects, indicating that the complicated interactions between 3+ parameters control the droplet field composition (color figure online)

While the violin plots in Fig. 6 establish the overall behavior of the droplet fields under different initial conditions, Sobol’ analysis can help identify the parameters controlling this behavior. The indices produced by Sobol’ analysis include the total effect of a parameter on the output ($S_T$), a value that encompasses both the primary impact of the indvidual parameter of interest ($S_1$) and influence of interactions between parameters on the QoI. Figure 7 shows the total effects ($S_T$, black) and the interaction effects ($S_T-S_1$, red) on the time of phase separation and the fraction of $K_1$ droplets for all initial conditions. The interactions between the parameters can be investigated even further through the $S_2$ value, which measures how the one-one interactions between two parameters impacts the QoI. Note that Sobol’ is one of the few methods that can quantify the interaction effects between parameters. Sobol’ can be further broken down into higher order interactions which, when taken together, describe and allocate the descriptive statistics of the sample distribution. It is typical to consider the total effects as this is correlated with other sensitivity measures. In particular, PRCC and the main effects in the method of Morris have very similar interpretations  (Saltelli and Annoni 2010). The higher order indices indicate interactions and can imply that specific parameter groupings play important roles. While this is often neglected when studies report the Sobol’ indices, these interactions can provide highly specific information about the nonlinear processes [3]. Figures 8, 9 and 10 explore the one-one parameter interactions ($S_2$) for each set of initial conditions. In these figures, the results from Fig. 7 are included for clarity, with the impact of the one-one parameter interactions in gray. All indices shown in Figures 7, 8, 9 and 10 were calculated using the final set of convergence evaluations (Table 2).

When examining the time of phase separation in systems with $1R_1:1R_2$ initial conditions, it becomes clear that the binding dynamics are the driving force. As shown in Fig. 7A, the Sobol indices for the total effects ($S_T$) of the binding dynamics on the time of phase separation are at least $4.6 \times$ higher than those for the $K_1$ and $K_2$ diffusion coefficients ($\lambda _{K_1}$,$\lambda _{K_2}$), signifying that it is the competition for the free protein between the two RNA complexes that controls the timescale on which phase separation emerges. Deconstructing $S_T$ shows that the total impact of each binding dynamic is a balance between its primary impact ($S_1$) and its interactions with other parameters, as evidenced by the interaction effects (red) that are roughly half of the $S_T$ indices (52–56$\%$ of the $S_T$ value).

Sobol’ analysis for the composition of the droplet field at the onset of separation with $1R_1:1R_2$ initial conditions is very different. While Fig. 7B shows that the fraction of $K_1$ droplets present are also primarily due to the binding dynamics and competition for free protein, the $K_1$ and $K_2$ diffusion coefficients now have a higher impact on this QoI, with the lowest $S_T$ value for the binding dynamics ($a_2$, $S_T = 0.392$) only $1.5 \times$ higher than for the $K_2$ diffusion coefficient ($S_T = 0.246$). Additionally, unlike the time of phase separation, for the fraction of $K_1$ droplets present at separation onset, the impact of interactions between the parameters accounts for much of the total influence each parameter has. As shown in Fig. 7B, the interaction effects comprise at least $68\%$ of the total effects, with the total effects for the $K_1$ binding parameters ($a_1$, $a_2$) due almost entirely to these interactions. Despite these differences, there is a commonality that does exist between the impact of the parameters on both the time of phase separation and the composition of the droplet field. Figure 8 shows the one-one parameter interactions ($S_2$) for the $1R_1:1R_2$ initial conditions. Here, we can see that the impact of the one-one interactions between each parameters, on both QoIs, make up a small fraction of the interaction effects, indicating that the interactions influencing both time of phase separation and the composition of the droplet field are due to the combined efforts of 3+ parameters, not direct interactions.

Skewing the initial conditions to favor $K_1$ droplet formation amplifies the patterns of parameter impact on the time of phase separation. For the $2R_1:1R_2$ initial conditions in Fig. 7C, MM screening reduced the parameter set to three binding dynamics ($a_1$, $a_2$, $a_4$) and the $K_1$ diffusion coefficient ($\lambda _{K_1}$). Just like the $1R_1:1R_2$ initial conditions, the binding dynamics impact the time of phase separation more than the diffusion coefficient. In particular, the most impactful parameters are the formation and disassociation of the $K_1$ complex ($a_1$, $a_2$). Both the $K_2$ disassociation rate ($a_4$) and the $K_1$ diffusion coefficient ($\lambda _{K_1}$) have a very small influence on the time of separation, with the $S_T$ values for $a_1$ and $a_2$ at least $4.6 \times$ higher. Additionally, with the $2R_1:1R_2$ systems, the interaction effects only make up $32\%$ of the total effects ($S_T$) of $a_1$ and $46\%$ of $a_2$, the two most influential parameters. But, unlike the $1R_1:1R_2$ initial conditions, the interaction effects of both parameters are mostly due to the one-one interaction with each other. As shown in Fig. 9A, B, the $S_2$ indices for the interaction account for roughly 70% of the interaction effects for both $a_1$ and $a_2$. These patterns continue when initial conditions are skewed even further towards $K_1$ formation. The reduced three-parameter set under $3R_1:1R_2$ initial conditions in Fig. 7E shows that the $a_1$ and $a_2$ parameters are responsible for the time of phase separation. Like the systems with the $2R_1:1R_2$ initial conditions, the impact of the $K_1$ associated binding dynamics is mostly due to the primary impact of these parameters, with the interaction effects comprising less than half of the $S_T$ value. Further, the contribution of these interaction effects on the time of phase separation is almost entirely due to the one-one interactions between the $a_1$ and $a_2$ parameters, as shown in Fig. 10A, B.

For the composition of the droplet field, the initial conditions skewed towards $K_1$ formation also produce relationships similar to those from the unbiased systems. As shown in Fig. 7D, F, Morris Method screening reduced the parameter set to five parameters for the $2R_1:1R_2$ initial conditions and three parameters for the $3R_1:1R_2$ systems. For these systems, while the total impact of the diffusion coefficient ($\lambda _{K_1}$) on the QoI is $S_T = 0.282$ for $2R_1:1R_2$ (7D) and $S_T = 0.32$ for $3R_1:1R_2$ (7F), the binding dynamics are the most impactful parameters on droplet field composition at this time point for both sets of initial conditions. However, unlike the time of phase separation in biased systems, it is not just the binding dynamics associated $K_1$ formation that are important. Rather, for the $2R_1:1R_2$ systems (7D), all four binding dynamics ($a_1-a_4$) have similar $S_T$ values ranging from $S_T = 0.401$ ($a_3$) to $S_T = 0.534$ ($a_4$). Investigation into the interaction effects for both skewed initial conditions showed that the total impact of all parameters involved in the fraction of $K_1$ droplets was due the influence of interactions between the parameters. As shown in Figs. 7D, F, the interaction effects comprise at least $48\%$ of the total effects ($S_T$) for all parameters. But, when the $S_2$ values are examined further in Fig. 9E–I for the $2R_1:1R_2$ systems and Fig. 10D–F for $3R_1:1R_2$ initial conditions, the $S_2$ indices measuring the impact of one-one interactions on the fraction of $K_1$ droplets were at most $48\%$ of the interaction effects. Thus, the composition of the droplet field observed at the onset of phase separation is most influenced by complex parameter dynamics that involve cooperative interactions between 3+ parameters.

**Fig. 11:**
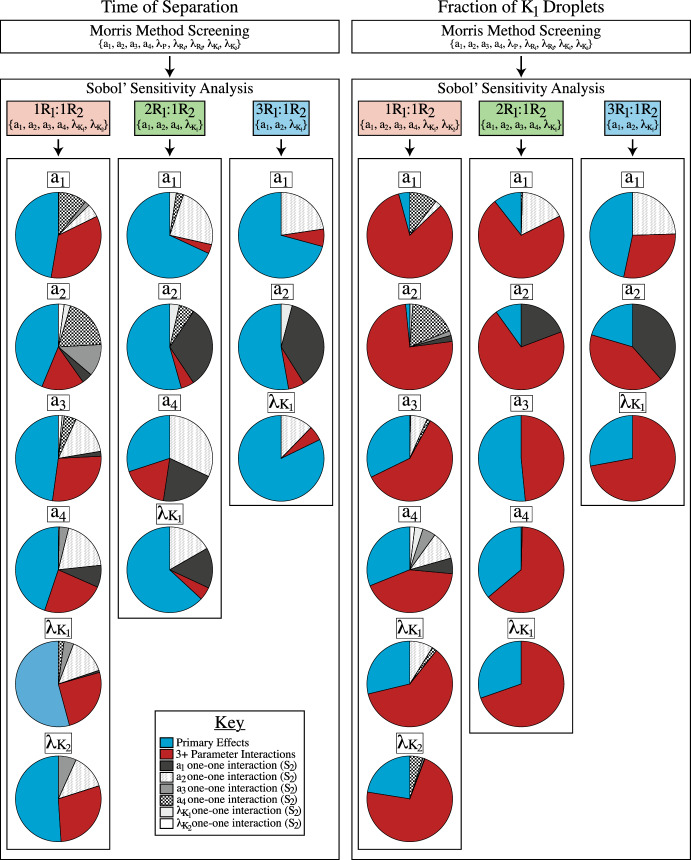
A summary of the combined sensitivity analyses performed and the complicated parameter influence revealed. For all initial conditions, performing Morris Method Screening reduces the parameter set for subsequent Sobol’ Sensitivity Analysis. Further, skewing the initial conditions towards $K_1$ formation reduces the parameter set for both outcomes to include only parameters associated with the formation and movement of $K_1$. Performing Sobol’ reveals intricate parameter interactions underlying observable LLPS phenomena. Here, each pie chart represents the total effects ($S_T$) of each parameter on the outcome, which is then broken down to show the percent of that effect that is attributed to the primary effects ($S_1$, blue), the one-one interaction with another parameter ($S_2$), and the higher order interactions (3+ parameters). It is clear that the primary effects ($S_1$) of each parameter are influential in determining the time of phase separation but complicated, higher-order interactions (3+ parameters) are controlling the composition of the droplet field at this time point (color figure online)

## Discussion

Examining liquid-liquid phase separation via sensitivity analysis is a logical choice given the complicated molecular dynamics at work. Sobol’ sensitivity analysis is a powerful tool for examining and quantifying the impact of both an individual parameter, as well as parameter interactions, on a given output. However, the ability to carry out Sobol’ is limited by the computational expense associated with it. Thus, preceding it with an initial sensitivity method gives the best of both worlds; a screening method can reduce the computational expense of Sobol’ by tailoring the parameter set to those of highest impact, and Sobol’ can then give an in depth look at the parameter interactions governing the QoI.

In this work, we examined intracellular phase separation under the lens of these combined methods. Originally proposed by Gasior et al., the mathematical model used here describes the competition between two RNA species for a shared protein-binding partner to drive phase separation. The authors of this previous work examined the role of RNA competition on the biophysical qualities associated with the droplet field by varying a few individual parameters, and ultimately highlighted the role of the binding dynamics in controlling droplet field properties. Here, we use the model as it was initially proposed, as well as systems subjected to skewed initial conditions. The model from Gasior et al. assumed the existence of well-mixed initial conditions that provided equal opportunity for each RNA species to bind with the protein molecules, allowing for true competition between the species. In addition to these $1R_1:1R_2$ initial conditions, we also bias the well-mixed initial conditions towards $K_1$ formation by increasing the initial concentration of $R_1$, an important component for $K_1$ formation. For each set of initial conditions, we analyzed the impact of the molecular dynamics on the time of phase separation and the composition of the droplet field at the onset of separation using a combination of Morris Method screening and Sobol’ sensitivity analysis. These outputs were chosen as they could, potentially, be measurable quantities in a wet lab environment with discrete values that do not depend on tracking transient changes to the droplet field. Further, dissecting the relationship between the molecular interactions and the resulting droplet properties is key to understanding how intracellular phase separation impacts cellular function. Together, these combined techniques revealed that the observed changes in phase separated systems are the result of complex interactions by multiple dynamics and intricate protein-RNA relationships, as shown in Fig. 11.

Sobol’ sensitivity analysis revealed the importance of protein-RNA interactions on the time of phase separation. For all three sets of initial conditions, Sobol’ indicated that the most impactful parameters were those associated with the binding and unbinding of the protein-RNA complexes. While both the $K_1$ and $K_2$ binding dynamics were deemed important with the $1R_1:1R_2$ initial conditions, skewing the initial conditions in favor of $K_1$ formation shifted parameter importance. Now, for the $2R_1:1R_2$ and $3R_1:1R_2$ initial conditions, parameters associated with $K_1$ formation are most influential the time of phase separation. These results indicate that it is the RNA competition for a shared resource that defines the timescale on which phase separation occurs. Under equal initial conditions, the stability and strength of the interactions impacts each protein-RNA species’ accumulation, subsequently determining how quickly the system can separate. Here, protein-RNA accumulation is a balance between all binding dynamics, and they must be accounted for to truly understand the complicated scope of protein competition that defines the time of phase separation. As the initial conditions are skewed, competition from the second RNA species ($R_2$) is lessened and the burden of separation falls on the formation and accumulation of the $K_1$ complex.

While the time of separation relied on protein-RNA interactions, the parameter interactions governing the droplet field composition reveal a dependence on spatial-temporal cooperation. Just like the time of phase separation, the fraction of $K_1$ droplets at the onset of separation is primarily impacted by the protein-RNA binding dynamics. The rate and strength of the molecular interactions defines how quickly each species can accumulate. However, unlike the time of separation, Sobol’ analysis revealed the protein-RNA complex diffusion coefficients have more of an effect on the fraction of $K_1$ droplets present at the onset of separation. Thus, it is not only the rate at which protein-RNA complexes accumulate in a well-mixed system, but how quickly like-complexes can find each other to form droplets. For the $1R_1:1R_2$ initial conditions, these results suggest spatial-temporal competition between the two RNA species is the driving force behind the composition. As each RNA species sequesters the free protein away from the other, it must retain it, at least until it can find like-complexes for droplet formation. Under initial conditions slightly biased towards $K_1$ formation ($2R_1:1R_2$), the competition persists as all of the protein-RNA interactions continue to have the highest impact. When the initial conditions are skewed further ($3R_1:1R_2$), the fraction of $K_1$ droplets depends on the rate of $K_1$ formation and coalescence. Thus, the droplet field composition at the onset of separation is due to the cooperation of several molecular dynamics, a relationship that is further hinted at through the 3+ parameter interactions controlling this QoI.

Despite the insights provided, there are still limitations and hidden dynamics underlying the results. One such dynamic is captured by the violin plots presented in Fig. 6B for the fraction of $K_1$ droplets at the onset of separation. Skewing the initial conditions from $1R_1:1R_2$ to $2R_1:1R_2$ shifts the density of these Sobol’ data sets from an approximately normal to a left-skewed distribution with most systems dominated by $K_1$ droplets. Additionally, MM screening shows a loss in distinction between the parameters deemed important for the fraction of $K_1$ droplets and those excluded from further Sobol’ analysis as the initial conditions changed from true competition to a system that favored $K_1$ formation. Thus, while not explicitly measured as part of these combined methodologies, the impact of the initial conditions is potentially the greatest indicator of the droplet field composition at the onset of separation.

Biased initial conditions had less impact on the time of phase separation. While Fig. 6A shows a shift in the distribution of the Sobol’ sampling data from a majority of systems separating at $t=3$s to $t=2$s, the median time remained at $t=3$s despite skewing the initial conditions. While these results could point to the robust control the binding dynamics have on the time of separation, it is possible that this lack of change is due, at least in part, to a fixed parameter in this model, $\chi _S$. Gasior et al. showed in their work that a higher value of $\chi _S$ leads to a faster time of phase separation for the system. With $\chi _S=4.25$, our systems will separate quickly, potentially producing the low and unchanging median ($t=3$s). Further, each set of samples had a small fraction that did not phase separate at all, despite having a high $\chi _S$ value, suggesting that it is the interactions between this demixing parameter and the parameters analyzed in this work that contribute to the observed timescale.

Despite the computational expense of Sobol’ analysis and the potential involvement of unseen parameters in both quantities of interest, this work provides insight into the complicated protein-RNA interactions controlling LLPS in the cellular environment. For temporal dynamics, such as the time of phase separation, the binding dynamics play an integral role. Conversely, spatial dynamics like the composition of the droplet field are more complicated, as they require intricate, high-level interactions from all parameters, including the binding dynamics, diffusion coefficients, and initial conditions. The dependence of both QoIs on the protein-RNA interactions revealed by Sobol’ indicates that Gasior et al. were not wrong in the conclusions of their analysis on RNA competition in LLPS phenomena, but that their work missed the complexity of the dynamics and was incomplete. However, we note that the single parameter analysis these authors performed was crucial as it clearly defined the role of the demixing parameters for this model, which could then be integrated into future work, making single parameter exploration the first step in detangling the impact of molecular dynamics on LLPS.

Our work ultimately establishes a quantitative framework for understanding the impact of molecular dynamics on intracellular phase separation. By combining multiple sensitivity analyses to reduce the parameter set, we can use Sobol’ analysis to investigate how parameters impact these biophysical traits of separation. This methodology can now be used moving forward to study the influence of changes to proteins and RNAs on transient dynamics that will evolve with the droplet population. Further, by highlighting the complicated and interwoven interactions underlying phase separation, these results offer caution when translating data into specific modeling parameters. This model is relatively small compared to the vast biological complexities that exist and so, attempts to correlate a single measurement with a single parameter could perpetuate false data and assumptions. Even simplifying the model or carrying out biological experiments between one RNA species and one protein species and attempting to link the time of phase separation with the mass action kinetics would, at minimum, miss the multivalent interactions possible between the molecules. Thus, we discourage the assumption that any one dynamic or model parameter can be discerned from a single measurement.

## Figures and Tables

**Fig. 12:**
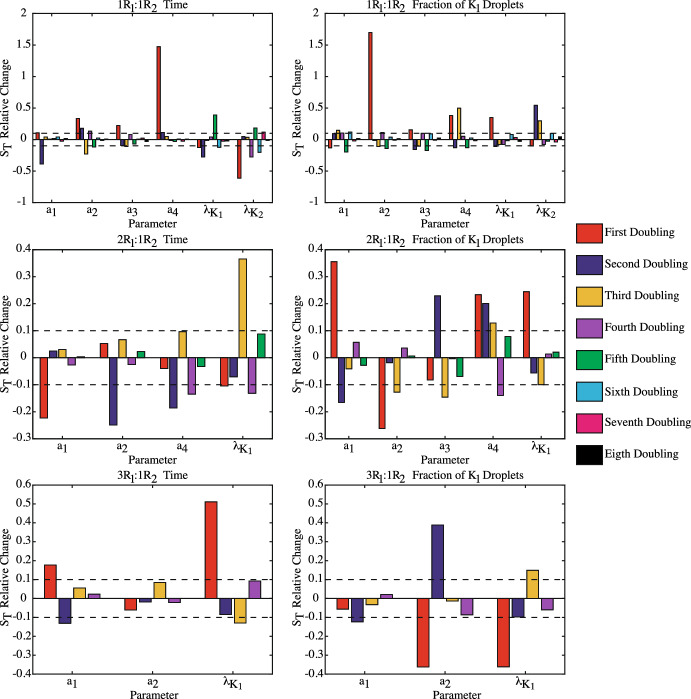
Convergence results for Sobol’ sensitivity analysis for both the time of phase separation and the fraction of $K_1$ droplets under all initial conditions ($1R_1:1R_2$, $2R_1:1R_2$, $3R_1:1R_2$). The smaller the subset of varied parameters, the fewer evaluations that were required for the Sobol’ indices to converge. For the $1R_1:1R_2$, six parameters were varied for both outputs, resulting in eight doublings and a final number of 114688 evaluations in Set 9. Skewing the initial conditions lowered the number of varied parameters and the total number of runs, with only three parameters varied under the $3R_1:1R_2$ and 8192 evaluations in Set 5 (color figure online)

## Appendix

**Table 2:** Percent of MM screening and Sobol’ analysis samples that did not phase separate

Initial	MM screening	% MM samples	Output	Final Sobol’	% Sobol’ evaluations
conditions	sample size	not separated		evaluation size	not separated
$1R_1:1R_2$	1000	17.6%	Time	114688	9.76%
			$K_1$ Fraction		
$2R_1:1R_2$	1000	15.5%	Time	20480	8.89%
			$K_1$ Fraction	24576	$9.02\%$
$3R_1:1R_2$	1000	14.3%	Time	8192	1.99%
			$K_1$ Fraction

**Table 3:** Number of Sobol’ evaluations for convergence

Set Number	$1R_1:1R_2$		$2R_1:1R_2$		$3R_1:1R_2$		Doubling
	Time Sep.	Fraction $K_1$	Time Sep.	Fraction $K_1$	Time Sep.	Fraction $K_1$	
	$(p = 6)$	$(p = 6)$	$(p = 4)$	$(p = 5)$	$(p = 3)$	$(p = 3)$	
Set 1	448		640	768	512		${-}{-}$
Set 2	896		1280	1536	1024		First
Set 3	1792		2560	3072	2048		Second
Set 4	3584		5120	6144	4096		Third
Set 5	7168		10240	12288	8192		Fourth
Set 6	14336		20480	24576	${-}{-}$		Fifth
Set 7	28672		${-}{-}$		${-}{-}$		Sixth
Set 8	57344		${-}{-}$		${-}{-}$		Seventh
Set 9	114688		${-}{-}$		${-}{-}$		Eighth

Under Morris Method Screening, all four binding dynamics ($a_1$, $a_2$, $a_3$, $a_4$) and all five diffusion coefficients ($\lambda _P$, $\lambda _{R_1}$, $\lambda _{R_2}$, $\lambda _{K_1}$, $\lambda _{K_2}$) are varied. Due to parameter combinations that could potentially produce slowly forming or weak protein-RNA complexes, as well as slow diffusion for any of the free protein, RNAs, or protein-RNA complexes, $< 20\%$ of the simulations for all three initial conditions did not phase separate by $t=1000$s. These systems were given a false time of separation $t = 1000$s and a false $K_1$ fraction of 1.25. The Sobol’ sampling method performed by  Herman and Usher (2017) on the reduced varied parameter sets produced fewer parameter regimes that failed to phase separate by $t=1000$s. Due to the convergence requirement for Sobol’ analysis, the time of phase separation was capped, with systems that had not separated by 10 seconds set to a time of $t = 10$s and a false $K_1$ fraction of 1.25. These systems comprised $< 10\%$ of the total evaluations performed in the final set. These results are detailed in Table 2.

To determine the $S_T$, $S_1$ and $S_2$ for each parameter, the number of simulations was doubled until $(S_{Ti_{2k}}-S_{Ti_k})/S_{Ti_k} < 0.1$ where *i* is the parameter and *k* is the number of simulations per set. Less than $10\%$ change in $S_T$ would indicate these statistics had stopped oscillating and converged to their true values. This method ensures that there are enough samples to conclude that our estimates have converged. Table 3 details the number of evaluations for each iteration of sampling the parameter space needed with Sobol’ analysis. The number of evaluations (*k*) for each set was calculated using the formula below where *n* is the number of Sobol’ samples and *i* is the number of varied parameters.

15 $$\begin{aligned} k = n \cdot (i + 2) \end{aligned}$$

For $1R_1:1R_2$ initial conditions, $i = 6$ parameters ($a_1$, $a_2$, $a_3$, $a_4$, $\lambda _{K_1}$, $\lambda _{K_2}$,) and thus Set 1 contained 448 evaluations (simulations). The number of total evaluations was doubled until the final set containing 114688 simulations. Figure 12A, B show the $S_T$ relative change between each set and the previous iteration, with the First Doubling indicating the relative change between $S_T$ values in Set 1 and Set 2.

For the $2R_1:1R_2$ initial conditions, 4 parameters ($a_1$, $a_2$, $a_4$, $\lambda _{K_1}$) were considered for time and 5 parameters ($a_1$, $a_2$, $a_3$, $a_4$, $\lambda _{K_1}$) were considered for fraction of $K_1$ droplets at the onset of phase separation. Using equation (A1), the initial set of Sobol’ evaluations for the time of separation included 640 simulations, while Set 1 for the fraction of $K_1$ droplets required 768. The number of evaluations required to reach convergence for these smaller parameter sets was much less than the $1R_1:1R_2$ initial conditions, with the time of separation requiring 20480 in Set 6, while the fraction of $K_1$ droplets required 24576. The reduced parameter set for $3R_1:1R_2$ required even fewer evaluations, with Set 5 (the final set) generating 8192 evaluations for both outputs.
